# Parasitic Nematodes Exert Antimicrobial Activity and Benefit From Microbiota-Driven Support for Host Immune Regulation

**DOI:** 10.3389/fimmu.2018.02282

**Published:** 2018-10-08

**Authors:** Sebastian Rausch, Ankur Midha, Matthias Kuhring, Nicole Affinass, Aleksandar Radonic, Anja A. Kühl, André Bleich, Bernhard Y. Renard, Susanne Hartmann

**Affiliations:** ^1^Department of Veterinary Medicine, Institute of Immunology, Freie Universität Berlin, Berlin, Germany; ^2^Bioinformatics Unit (MF 1), Robert Koch Institute, Berlin, Germany; ^3^Core Unit Bioinformatics, Berlin Institute of Health (BIH), Berlin, Germany; ^4^Berlin Institute of Health Metabolomics Platform, Berlin Institute of Health (BIH), Berlin, Germany; ^5^Max Delbrück Center for Molecular Medicine, Berlin, Germany; ^6^Centre for Biological Threats and Special Pathogens (ZBS 1), Robert Koch Institute, Berlin, Germany; ^7^Genome Sequencing Unit (MF 2), Robert Koch Institute, Berlin, Germany; ^8^iPATH.Berlin, Core Unit for Immunopathology for Experimental Models, Berlin Institute of Health, Charité - Universitätsmedizin Berlin, Corporate Member of Freie Universität Berlin, Humboldt-Universität zu Berlin, Berlin, Germany; ^9^Institute for Laboratory Animal Science, Hannover Medical School, Hannover, Germany

**Keywords:** parasite, nematode, immune regulation, germ-free, microbiota, antimicrobial, Treg, Th2

## Abstract

Intestinal parasitic nematodes live in intimate contact with the host microbiota. Changes in the microbiome composition during nematode infection affect immune control of the parasites and shifts in the abundance of bacterial groups have been linked to the immunoregulatory potential of nematodes. Here we asked if the small intestinal parasite *Heligmosomoides polygyrus* produces factors with antimicrobial activity, senses its microbial environment and if the anti-nematode immune and regulatory responses are altered in mice devoid of gut microbes. We found that *H. polygyrus* excretory/secretory products exhibited antimicrobial activity against gram^+/−^ bacteria. Parasites from germ-free mice displayed alterations in gene expression, comprising factors with putative antimicrobial functions such as chitinase and lysozyme. Infected germ-free mice developed increased small intestinal Th2 responses coinciding with a reduction in local Foxp3^+^RORγt^+^ regulatory T cells and decreased parasite fecundity. Our data suggest that nematodes sense their microbial surrounding and have evolved factors that limit the outgrowth of certain microbes. Moreover, the parasites benefit from microbiota-driven immune regulatory circuits, as an increased ratio of intestinal Th2 effector to regulatory T cells coincides with reduced parasite fitness in germ-free mice.

## Introduction

Infections with enteric nematodes are associated with changes in the composition of the host intestinal microbiota in mice, pigs, and primates (1–5). Our previous work showed that nematode-infected mice deficient in IL-4Rα-signaling, hence refractory to IL-4/IL-13-dependent immune sequelae, experience similar microbiota alterations as fully immune-competent mice (2), leaving open the question of the mechanistic basis for structural changes in microbial communities associated with nematode infections. Our and other groups have shown that products released by parasitic nematodes possess antimicrobial activity (6–8), prompting the question if enteric nematodes sense and actively shape their microbial environment.

To ensure prolonged survival and reproduction, parasitic nematodes have developed strategies suppressing host immune responses, in part driven by the release of immunomodulators interfering with innate and adaptive immune effector mechanisms (9–11), but also by supporting the *de novo* generation, expansion and activation of regulatory T cells (Treg) (12–16). Recent studies provide evidence for a contribution of microbiota alterations to immune regulation during nematode infection. More specifically, the increased abundances of Lactobacilli and Clostridiales family members during nematode-infection have been linked to the expansion and activation of Treg (1, 17), which in turn control the magnitude of anti-parasite and unrelated inflammatory responses (13–16, 18).

Here we focused on the interaction of an enteric parasite infection, microbiota, and host immunity. We surveyed fitness and gene expression of the small intestinal nematode *Heligmosomoides polygyrus* reared in conventional and germ-free mice and investigated products released by the parasite for antimicrobial activity against gram^−^ and gram^+^ bacterial species. Furthermore, we compared anti-parasite Th2 immunity and the expansion, cytokine production and phenotypic heterogeneity of Treg in conventional and germfree mice. Our data demonstrate that (I) *H. polygyrus* may actively shape the composition of the host microbiota by releasing antimicrobials and that (II) nematode fitness is compromised in the absence of host microbes. Furthermore, our data suggest that the nematode senses the microbiota, as indicated by differential gene expression of worms from germ-free and conventional hosts, and finally, that microbes support Treg responses regulating anti-parasite Th2 immunity.

## Materials and methods

### Mice and parasites

The experiments performed followed the National Animal Protection Guidelines and were approved by the German Animal Ethics Committee for the protection of animals (G0176/16). Female specific pathogen-free (SPF) and germfree C57BL/6 mice were kept in individually ventilated, filter-topped cages with autoclaved bedding, chow and water. Infections with 200 *H. polygyrus* larvae were performed aseptically in a laminar flow. *H. polygyrus* L3 were freshly isolated from fecal cultures of infected mice and treated for 1 week with an antibiotic cocktail (5 mg/ml streptomycin, 1 mg/ml ampicillin, 0.5 mg/ml gentamicin, 1 mg/ml neomycin, 0.5 mg/ml vancomycin; all from AppliChem, Darmstadt, Germany). L3 were shown to be free of aerobic microbes as determined by lack of bacterial growth in antibiotic-free LB medium. Infected and naïve control GF mice received antibiotics (as specified above) via the drinking water. To further reduce the risk of contamination, SPF and GF C57BL/6 mice were kept without bedding change until the dissection 2 weeks post-infection. The axenic status of GF mice was confirmed by qPCR of eubacterial 16s rRNA with colon content collected on the day of infection and dissection. Adult worms were removed from the small intestine, counted and eight females per mouse were kept at 37°C in RPMI-1640 medium containing 200 U/mL penicillin, 200 μg/mL streptomycin (all from PAN Biotech, Aidenbach, Germany) and 1% glucose for 24 h for the determination of individual egg counts. Female worm length was determined after culture.

### Parasite excretory/secretory products

Excretory/secretory products of *H. polygyrus* (HES) were collected from adult worms extensively washed before being cultured in phenol-red free RPMI-1640 medium containing 200 U/mL penicillin, 200 μg/mL streptomycin. After 24 h in culture, worms were washed extensively with antibiotic-free worm growth media (RPMI-1640 medium with 1% glucose) and maintained in this medium with daily media changes. Spent media from the first 48 h were discarded. Thereafter, supernatants were harvested every 48 h and sterile filtered through a 0.22 μm syringe-driven filter system, and stored at −20°C until further use.

### Bacterial strains

The strains used to evaluate antibacterial activities of HES in the radial diffusion assay included *Escherichia coli* IMT19224, *Salmonella enterica* serovar Typhimurium ATCC14028, and *Staphylococcus aureus* IMT29828 obtained from the strain collection of the Institute of Microbiology and Epizoonotics, Freie Universität Berlin and *Enterococcus faecium* DSM20477 provided by Dr. Markus Heimesaat (Institute of Microbiology, Charité—Universitätsmedizin Berlin). *E. coli* IMT19224 was used to assess agglutinating activity of HES.

### Radial diffusion assay

Antibacterial activities of HES were assessed using the radial diffusion assay (19). Overnight bacterial cultures were diluted 1:100 in Mueller-Hinton broth (Carl Roth, Karlsruhe, Germany) and incubated at 37°C with shaking at 250 rpm until reaching an optical density of 0.3–0.4 at 600 nm. Bacteria were washed and resuspended in cold sodium phosphate buffer (100 mM, pH 7.4) by centrifugation (880 × g, 10 min, 4°C). Bacteria were then resuspended in warm (50°C), sterile underlay agar [10 mM sodium phosphate buffer, 1% (v/v) Mueller-Hinton broth, 1.5 (w/v) agar] at 4 × 10^5^ colony forming units per mL. Fifteen milliliter of bacteria-infused underlay agar was poured into 120 mm square petri dishes and allowed to solidify. Evenly spaced wells (5 mm) were formed in the agar using the blunt ends of P10 pipet tips, and treatments and controls added (5 μL/well). Five microliter native HES corresponded to 5 μg protein. The antimicrobial peptide Pexiganan (kindly provided by Jens Rolff, Institute of Biology, Freie Universität Berlin, 0.0125 μg/well) was applied as positive control. PBS and RPMI-1640 medium were included as negative controls. Plates were incubated at 37°C for 3 h and then overlaid with double-strength Mueller-Hinton agar [4.2% (w/v) Mueller-Hinton broth, 1.5% agar]. Petri dishes were incubated for 18 h at 37°C and the growth inhibition zones around each well were measured. Antibacterial activity is represented as the diameter of the inhibition zone (mm) beyond the 5 mm well.

### Agglutination assay

Agglutinating activity of HES was assessed as described previously (20) using *E. coli* IMT19224. Bacteria were collected at mid-logarithmic phase by centrifugation at 880 × g for 5 min, then washed and resuspended in Tris-buffered saline (50 mM Tris-HCl, 150 mM NaCl, pH 7.5) at approximately 10^9^ cells/mL. Thirty microliter of bacteria were mixed with 30 μL of treatments in the presence and absence of 10 mM CaCl_2_ and incubated for 1 h at room temperature on a glass slide. Concanavalin A from *Canavalia ensiformis* (Con A) and Lectin from *Triticum vulgaris* (Wheat germ agglutinin; WGA, both from Sigma-Aldrich) were included as positive controls. Samples were then visualized and photographed using the 40X objective on a Leica DM750 microscope equipped with an ICC50HD digital camera (Leica Microsystems, Wetzlar, Germany).

### Parasite RNA-isolation and quality check

Small intestines and the bulk of removed parasites were kept in ice-cold physiological NaCl solution. Thirty worms (15 males/15 females) were quickly isolated from three individual SPF and GF mice, washed repeatedly in cold physiological NaCl solution, inspected for physical integrity, and absence of host tissue and then snap frozen in liquid nitrogen before storage at −80°C. Samples were homogenized using shredder columns filled with 200 mg sterile sea sand and the FastPrep®-24 instrument (MP Biomedicals, Eschwege, Germany) at 5 m/s for 35 s. Supernatants of homogenized worms were further processed for RNA isolation (InnuPREP RNA isolation, Analytik Jena AG, Germany), DNase treatment (Analytik Jena AG, Germany), and RNA quality control (Agilent 2100 Bioanalyzer, RNA 6000 Nano Kit, Agilent Technologies, Waldbronn, Germany). All RNA samples displayed RIN values of 10.

### Sequencing and data processing

For transcriptome sequencing on an Illumina platform a TruSeq RNA library generation was utilized. The library was generated by using the TruSeq RNA Sample Prep Kit v2 (Illumina, San Diego, CA, USA) following the manufacturer's instructions. The library was quantified by using the KAPA Library Quantification Kit for Illumina (Kapa Biosystems, Wilmington, MA, USA). The library size was determined by using the High Sensitivity DNA Analysis Kit for the 2100 Bioanalyzer Instrument (Agilent Technologies, Waldbronn, Germany). Libraries were adjusted to a concentration of 12 pM and sequenced on a HiSeq 1500 instrument (Illumina, San Diego, CA, USA) in rapid mode. For cluster generation, the TruSeq Rapid PE Cluster Kit v2 was used. Cluster generation was performed on board. For sequencing the HiSeq Rapid SBS kit v2 was used to sequence 100 + 100 bases.

We sequenced three isolates from SPF and GF mice with a mean library size of 40.15 million paired-end reads and a standard deviation of 10.74. Raw reads were subjected to quality control and trimming via the QCumber pipeline (version 1.0.14, https://gitlab.com/RKIBioinformaticsPipelines/QCumber) utilizing FastQC (v0.11.5, https://www.bioinformatics.babraham.ac.uk/projects/fastqc/), Trimmomatic (0.36) (21) and Kraken (0.10.5-beta) (22). On average, 91.77% of reads remained after trimming.

Preprocessed reads were mapped to a reference genome (as specified below) and corresponding sequence features using the TopHat split-read mapper (v2.1.1) (23) and reference as well as novel features were extracted and merged with the aid of Cufflinks and Cuffmerg (24) (v2.2.1) to obtain one integrated and unified transcriptome for *H. polygyrus* samples. The *H. polygyrus* draft genome nHp_v2.0 was applied as reference genome (database version WBPS10, annotation version 2016-09-WormBase), as available at WormBase ParaSite (25). For each sample, raw expression values were created by counting uniquely mapped reads on gene level using featureCounts (v1.5.0-p3) (26). To identify differentially expressed genes (DEGs) between SPF and GF mice isolates, respectively, DESeq2 (1.12.4) (27) was applied with a classic pairwise design model and a *p*-value threshold of 0.05. In addition, normalized and transformed expression values were extracted from DESeq2 (regularized log transformation) and corrected for batch effects via Limma (3.28.21, removeBatchEffect) (28) to allow for sample comparison with clustered heatmaps and principal component analysis (PCA).

Reference as well as novel transcripts were functionally (re-)annotated using an iterative annotation strategy. First, transcripts were either first-frame translated (reference) or examined for ORFs (novels, Cuffcompare class code “u”) using EMBOSS transeq (6.6.0.0) (29) and TransDecoder (v2.1), respectively. Next, resulting protein sequences were passed through a series of database searches until successfully annotated with Gene Ontology (GO) terms (30), either via blastp (2.6.0+) (31), and Blast2GO (4.0.7) (32) or by a final InterProScan (33). Databases used for annotation included (in this order) the UniProt (34) *Heligmosomoides polygyrus bakeri* proteome (UP000050761, downloaded at 07.04.2017), UniProt Swiss-Prot Nematoda proteins, UniProt TrEMBL Nematoda proteins as well as the complete Swiss-Prot database and the complete TrEMBL database (all downloaded at 16.02.2017).

### Cell isolation, stimulation, and flow cytometry

Lymph node single cell suspensions and small intestinal tissue digestion for the isolation of siLP cells were performed as described previously (35). Cultures were kept for 6 h with brefeldin A added after 1 h before surface and intracellular staining. Surface and intracellular markers were stained according to the manufacturer's instructions with the following antibodies obtained from ThermoFisher/eBioscience, if not stated otherwise: CD4-PerCP/-BV510/-A700 (RM4-5), Foxp3-FITC/-PerCP-Cy5.5 (FJK-16s), GATA-3-A660/-PE/-PE-eF610 (TWAJ), T-bet-PE/-PE-Cy7 (eBio4B10), RORγt-BV421 (Q31-378, BD biosciences), IL-10-APC (JES5-16E3), IL-4-PE/-PE-Cy7 (11B11), and IL-17A-PerCP-Cy5.5 (eBio17B7). Live/dead discrimination was performed using fixable viability dye eF780 (ThermoFisher/eBioscience). Unspecific binding was prevented by addition of 20 μg/ml FcgRII/III blocking antibody (2.4G2).

### Histology

Formalin-fixed, paraffin-embedded sections (1–2 μm) of duodenum were de-waxed and stained with hematoxylin and eosin for overview, with periodic acid Schiff for goblet cell quantification and by Direct red 80 (Sigma) for the detection of eosinophils. Enteritis was scored using hematoxylin and eosin-stained section as described before (16). PAS^+^ goblet cells were counted along five villi per section. Images were acquired using the AxioImager Z1 microscope (Carl Zeiss MicroImaging, Inc., Göttingen, Germany). All evaluations were performed blinded.

### Statistical analyses

Data were assessed for normality using GraphPad Prism software (La Jolla, CA, USA). For comparison between two groups, an unpaired *T*-test was used. Testing of multiple groups was performed using a one-way analysis of variances followed by Tukey's multiple comparison or the Kruskall-Wallis test combined with Dunn's multiple comparison test.

## Results

### Antimicrobial activity of nematode excretory/secretory products

Infection with *H. polygyrus* alters the composition of the intestinal microbiota alongside the intestine, including an increase in gram^−^ Enterobacteriaceae (2, 17, 36). Similar changes occurred in IL-4Rα^−/−^ mice, hence independently of Th2-mediated changes in gut physiology (2). As both free-living and parasitic nematodes defend themselves against potentially harmful microbes by the production of antimicrobial factors (7), we asked if *H. polygyrus* releases active antimicrobials, possibly interfering with its microbial environment. We used the radial diffusion assay to test the antibacterial activity of *H. polygyrus* excretory/secretory products (HES) in comparison to the antimicrobial peptide Pexiganan. Five micrograms of native HES collected from *H. polygyrus* cultures inhibited the growth of gram^−^ and gram^+^ bacteria, including *E. coli, S. enterica* var. Typhimurium, *E. faecium*, and *S. aureus* (Table 1).

**Table 1:** Antimicrobial activity^*^ of excretory/secretory products from adult *Heligmosomoides polygyrus* nematodes in the radial diffusion assay.

	***E. coli***	***S. typhimurium***	***E. faecium***	***S. aureus***
	**IMT19224**	**ATCC14028**	**DSM20477**	**IMT29828**
*H. polygyrus* E/S (5 μg)	5.3 ± 3.1	4.3 ± 0.6	3.7 ± 1.5	5.7 ± 1.5
Pexiganan (0.0125 μg)	9.0 ± 0.0	8.0 ± 0.0	12.0 ± 0.0	13.0 ± 0.0
PBS	–	–	–	–
RPMI-1640	–	–	–	–

* *Activity reported as inhibition zone (mm; mean ± standard deviation) produced by 5 μL treatments (n = 3 biological replicates with independent batches of HES). “–”indicates no detectable activity. Data are representative for two independent experiments*.

**Figure 1:**
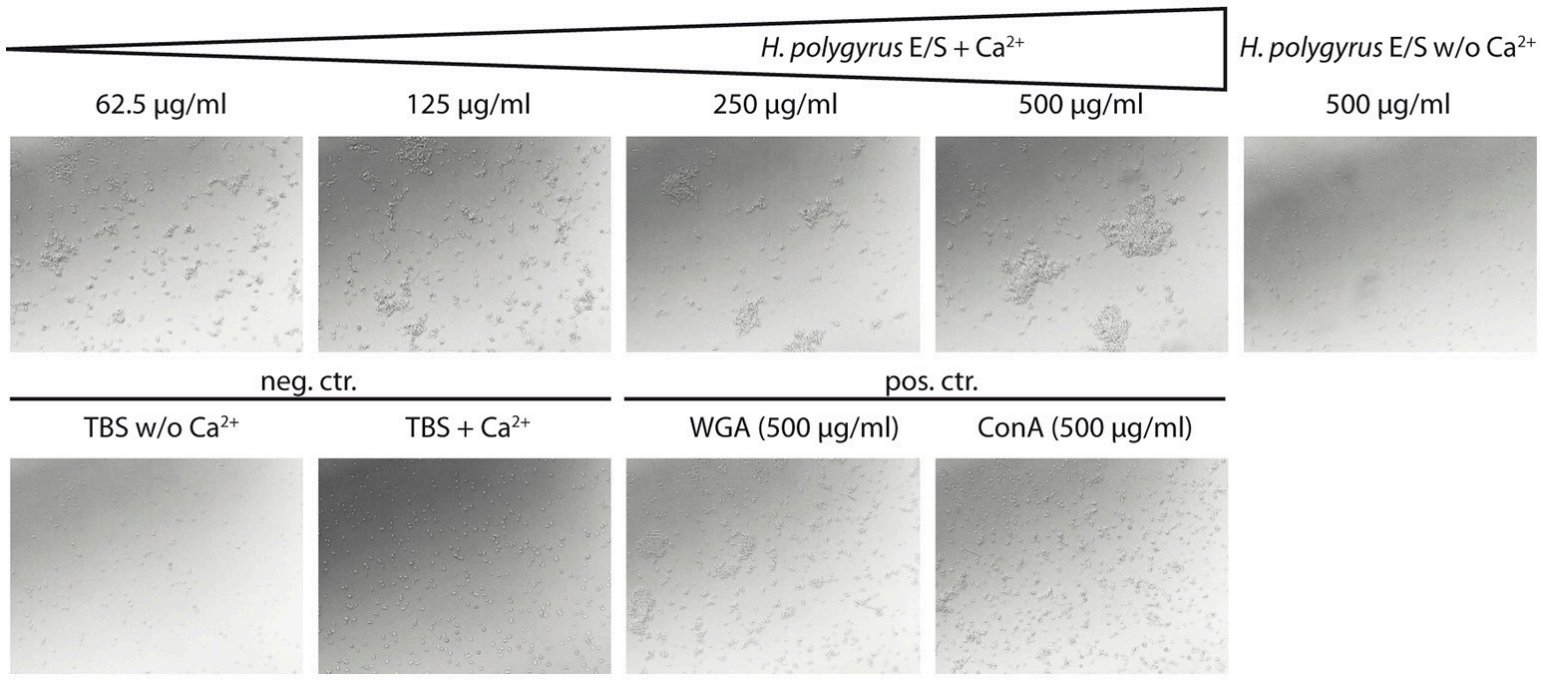
*H. polygyrus* excretory/secretory products cause bacterial agglutination. **(Top)** Bacterial agglutination in the presence and absence of native adult *H. polygyrus* E/S products (HES) and 10 mM CaCl_2_. Representative images of agglutination of *E. coli* IMT19224 with serial dilutions of *H. polygyrus* E/S products are shown. **(Bottom)** controls of agglutination include tris-buffered saline (TBS) with and without CaCl_2_ as well as the C-type lectins wheat germ agglutinin (WGA) and concanavalin A (Con A). Magnification x400. Data are representative for two individual experiments performed with two independent HES batches.

C-type lectin domain-containing proteins are known to agglutinate bacteria and are important in nematode immune defense against microbial infection (37). As *H. polygyrus* produces a C-type lectin protein (38) we tested the agglutinating activity of nematode products by treating *E. coli* with increasing amounts of native HES in the presence and absence of CaCl_2_. We observed dose- and calcium-dependent agglutinating activity (Figure 1), suggestive of C-type lectin-mediated bacterial agglutination. These data indicate that *H. polygyrus* employs defense mechanisms via released products during its interactions with microbes which may contribute to shaping its microbial environment in the murine gut.

### Altered parasite gene expression in germ-free mice

**Figure 2:**
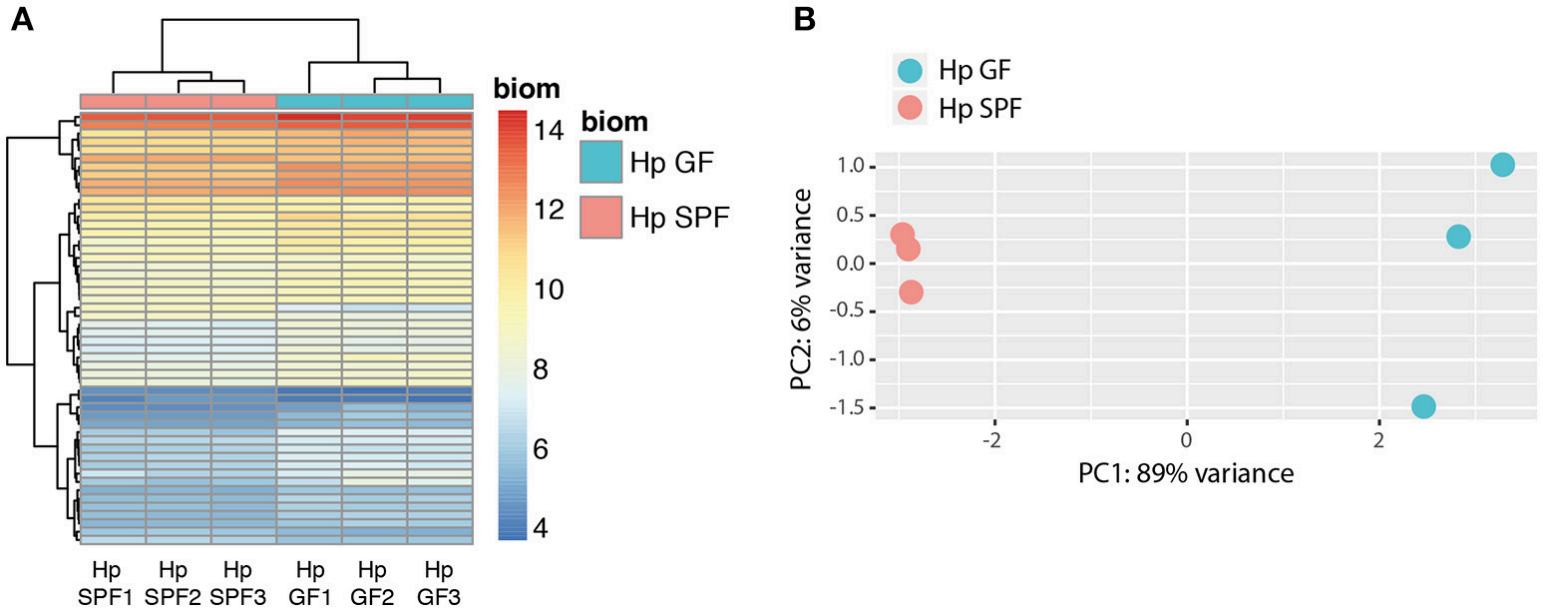
Principle component analysis (PCA) and clustering of differentially expressed genes (DEGs). **(A)** Unsupervised clustering heatmap of differentially expressed genes (DEG, *n* = 52) in *H. polygyrus* samples isolated from SPF and GF mice. Red intensity indicates high gene expression, whereas blue intensity indicates low gene expression. **(B)** Principle component (PC) analysis revealed that 89% of the data variation is explained by the difference between SPF and GF isolates. Data are from one experiment with three biological replicates.

Having demonstrated the ability of nematode products to influence bacterial growth, we sought to investigate if intestinal nematodes sense their microbial environment and hence asked if the complete absence of microbes in the host gut resulted in altered parasite gene expression. To that end, we infected germfree (GF) and conventional (specific pathogen-free; SPF) mice and performed RNA-sequencing with parasites isolated 2 weeks post-infection. Samples clearly clustered according to SPF vs. GF parasite origin (Figures 2A,B). We found that a surprisingly small set of 52 genes was differentially expressed in adult worms isolated from GF compared to SPF mice (Supplementary Table 1). The majority of genes were upregulated, comprising a venom-like allergen (VAL-1), chitinase-1, lysozyme-3, and orthologs of putative *Caenorhabditis elegans* glutathione S-transferase and *C. elegans*/*C. briggsae* UDP-glucuronosyl- transferases, amongst others (Supplementary Table 1). Only four of ten genes downregulated in parasites isolated from GF mice were annotated, including a putative *C. elegans* UDP-glucuronosyl-transferase. Hence, parasitic nematodes reared in a germ-free environment display a distinct gene expression pattern.

### Reduced parasite fitness in germ-free mice

**Figure 3:**
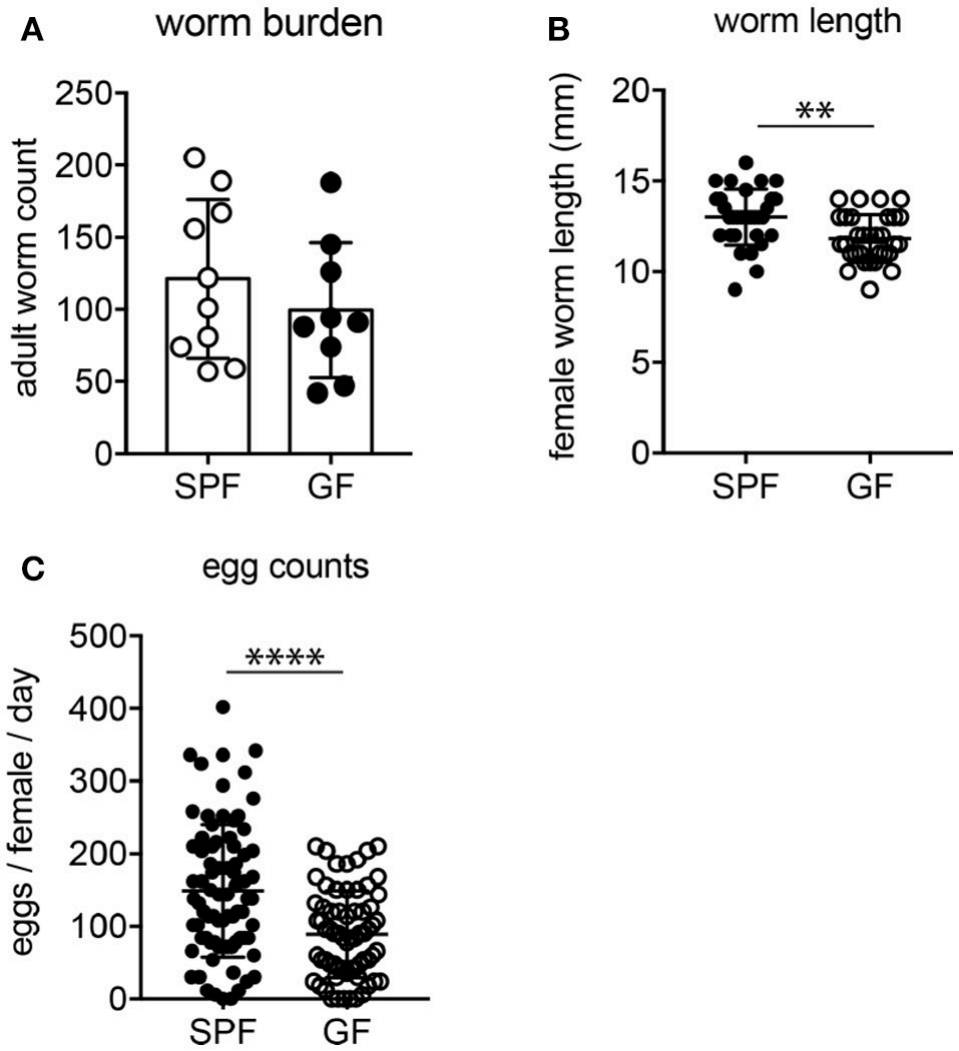
Parasite burden and fitness in SPF and GF mice. **(A)** Number of luminal adults isolated 2 weeks post-*H. polygyrus* infection from SPF and GF mice. **(B)** Length of female parasites. **(C)** Fecundity of female worms determined as egg production within 24 h after isolation. Data are pooled from two independent experiments each performed with four to five infected mice per group. Mean, SD, and individual data points are shown. ^**^p < 0.01, ^****^p < 0.0001.

Previous studies reported on impeded survival and fecundity of intestinal nematodes in the absence of gut microbes (39–41); therefore, we assessed if parasite burden and fitness were altered depending on the host microbial status. While adult worm burdens were similar in SPF and GF mice at 2 weeks post-infection (Figure 3A), female worms developing in GF mice were significantly smaller and produced fewer eggs (Figures 3B,C). Importantly, *H. polygyrus* resides in the proximal small intestine harboring few microbes and the parasite mainly relies on host tissue as food source (42). Thus, we investigated next if the reduced parasite fitness in GF mice coincided with immune changes.

### Altered treg responses in nematode-infected germ-free mice

The microbiota supports the induction and maintenance of regulatory T cells (Treg) (43–46) and infections with *H. polygyrus* lead to the activation and expansion of regulatory T cells suppressing local immunopathology, but also host protective Th2 immunity (1, 15–17). Therefore, we surveyed if Treg expansion, phenotype and cytokine production in *H. polygyrus* infected mice differed depending on the microbial status.

**Figure 4:**
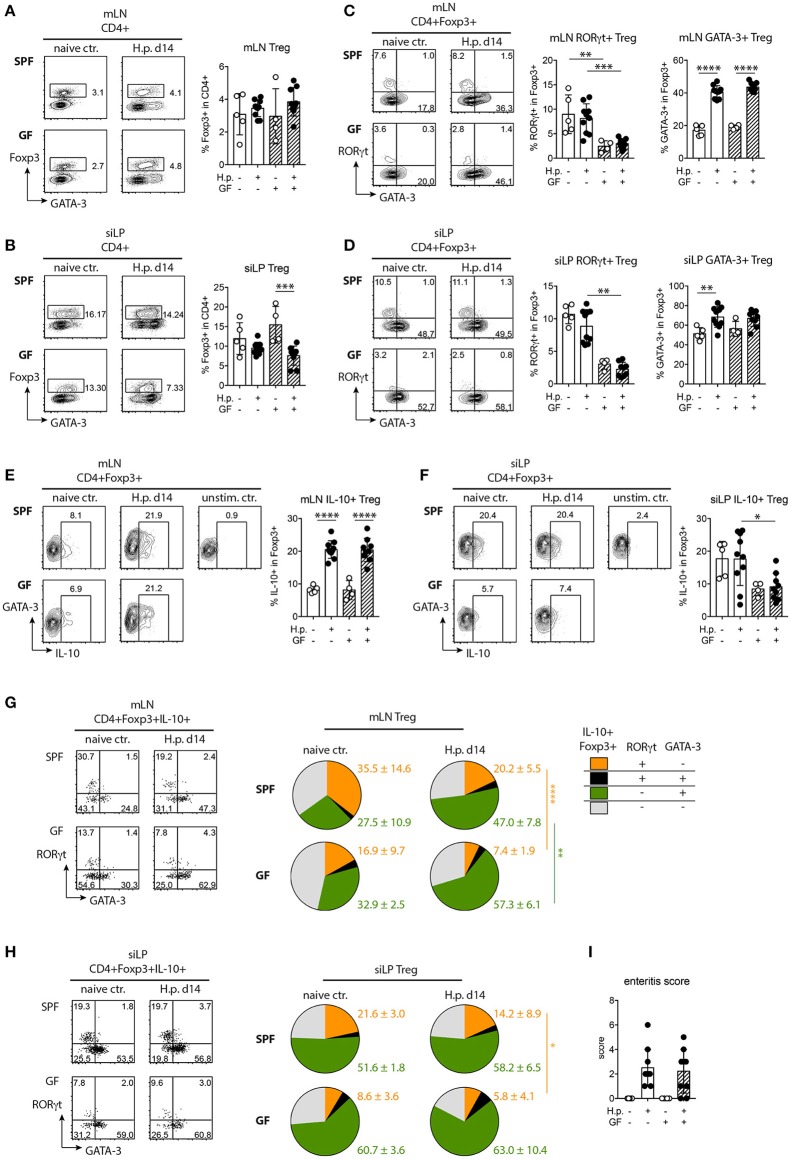
Treg responses in SPF and GF mice infected with *H. polygyrus*. **(A,B)** Representative plots of Foxp3^+^ Treg detection in CD4^+^ T cells and Treg frequencies in mesenteric lymph nodes (mLN, **A**) and small intestinal lamina propria (siLP, **B**) of uninfected controls and mice infected with *H. polygyrus* for 2 weeks. **(C,D)** Representative plots of RORγt and GATA-3 expression by Treg and frequencies of RORγt^+^ and GATA-3^+^ Treg in mLN **(C)** and siLP **(D)**. **(E,F)** Representative plots of IL-10 expression and frequencies of IL-10^+^ Treg in mLN **(E)** and siLP **(F)**. **(G,H)** Representation of RORγt^+^, RORγt^+^GATA-3^+^, GATA-3^+^, and RORγt^−^GATA-3^−^ Treg in the IL-10^+^ Treg population in mLN and siLP of naïve and infected mice. Numbers express group means and SD. **(I)** Duodenal enteritis scores. Data are pooled from two independent experiments each performed with two to three uninfected and four to five infected mice per group. Mean, SD, and individual data points are shown in **(A–H)**. ^*^p < 0.05; ^**^p < 0.01, ^***^p < 0.001, ^****^p < 0.0001.

The overall frequencies of Foxp3^+^ Treg were similar in mLN of naive SPF and GF mice and did not change significantly upon infection (Figure 4A). While Treg frequencies in the small intestinal lamina propria (siLP) were stably maintained in infected SPF mice, Treg frequencies dropped significantly in the small intestines of infected GF mice compared to the respective naive controls (Figure 4B).

Intestinal Foxp3^+^ Treg form a functional heterogeneous population comprising subsets marked by the elevated expression of GATA-3 or RORγt, respectively (47). While GATA-3 expression is necessary for Treg stability under inflammatory conditions (48, 49), RORγt^+^ Treg exhibit a highly activated phenotype and limit the Th2-driven control of helminth infection and immune pathology in intestinal inflammation (43, 50). Hence, we investigated if the reduced fitness of worms isolated from GF mice was associated with phenotypic alterations in the Treg population. Fewer Treg in mLN and siLP of naïve and infected GF mice expressed RORγt compared to the respective SPF controls (Figures 4C,D). Steady state GATA-3 expression by Treg and the expansion of GATA-3^+^ Treg upon infection was similar in mLN of SPF and GF mice (Figure 4C). Upon infection, the increase in GATA-3^+^Treg reached significance in the small intestine of SPF mice (Figure 4D). Thus, naïve and infected GF mice harbored significantly less RORγt^+^ Treg compared to SPF mice, while GATA-3^+^Treg expanded similarly.

Next, we asked if Treg activation differed depending on the microbial status and hence assessed their cytokine production. IL-10 production by Treg in mLN increased similarly and strongly in SPF and GF mice upon infection (Figure 4E). IL-10 production by siLP Treg of SPF mice did not change in response to infection (Figure 4F). Small intestinal Treg of GF mice were rather poor IL-10 producers at steady state and upon infection (Figure 4F). As intestinal RORγt^+^ Treg have been reported as superior in IL-10 production compared to other gut Treg (43), we next surveyed IL-10^+^ Treg of SPF and GF mice for co-expression of RORγt and GATA-3. Expectedly, the reduced frequencies of RORγt^+^ cells in the Foxp3^+^Treg pool (Figures 4C,D) was reflected by their underrepresentation in the IL-10 producing Treg population of naïve and infected GF mice (Figures 4G,H). GATA-3^+^Treg expanding in mLN of infected SPF and GF mice (Figure 4C) clearly dominated the IL-10-expressing Treg pool in both groups upon infection (Figure 4G). Reflecting their high frequencies in the total siLP Treg population (Figure 4D), GATA-3^+^Treg dominated in the small intestinal IL-10^+^ population irrespective of microbial and nematode-infection status (Figure 4H). Finally, we investigated if the reduction in RORγt^+^ Treg in the intestine and the poor IL-10 expression by gut Treg in GF mice was associated with differences in local immunopathology. Duodenal enteritis scores were, however, similar in SPF and GF nematode-infected mice (Figure 4I).

Taken together our data show that Treg activation in gut-associated lymphoid tissue seen as increased IL-10 production occurs independently of the presence of gut microbes. Naïve and nematode-infected GF mice display a reduction in RORγt^+^ Treg in the gut and gut-draining lymph nodes, while GATA-3^+^ Treg expanded similarly in SPF and GF mice and formed the major IL-10 producing Treg subset upon infection irrespective of the microbial status.

### Increased Th2/Treg ratios in nematode-infected germ-free mice

**Figure 5:**
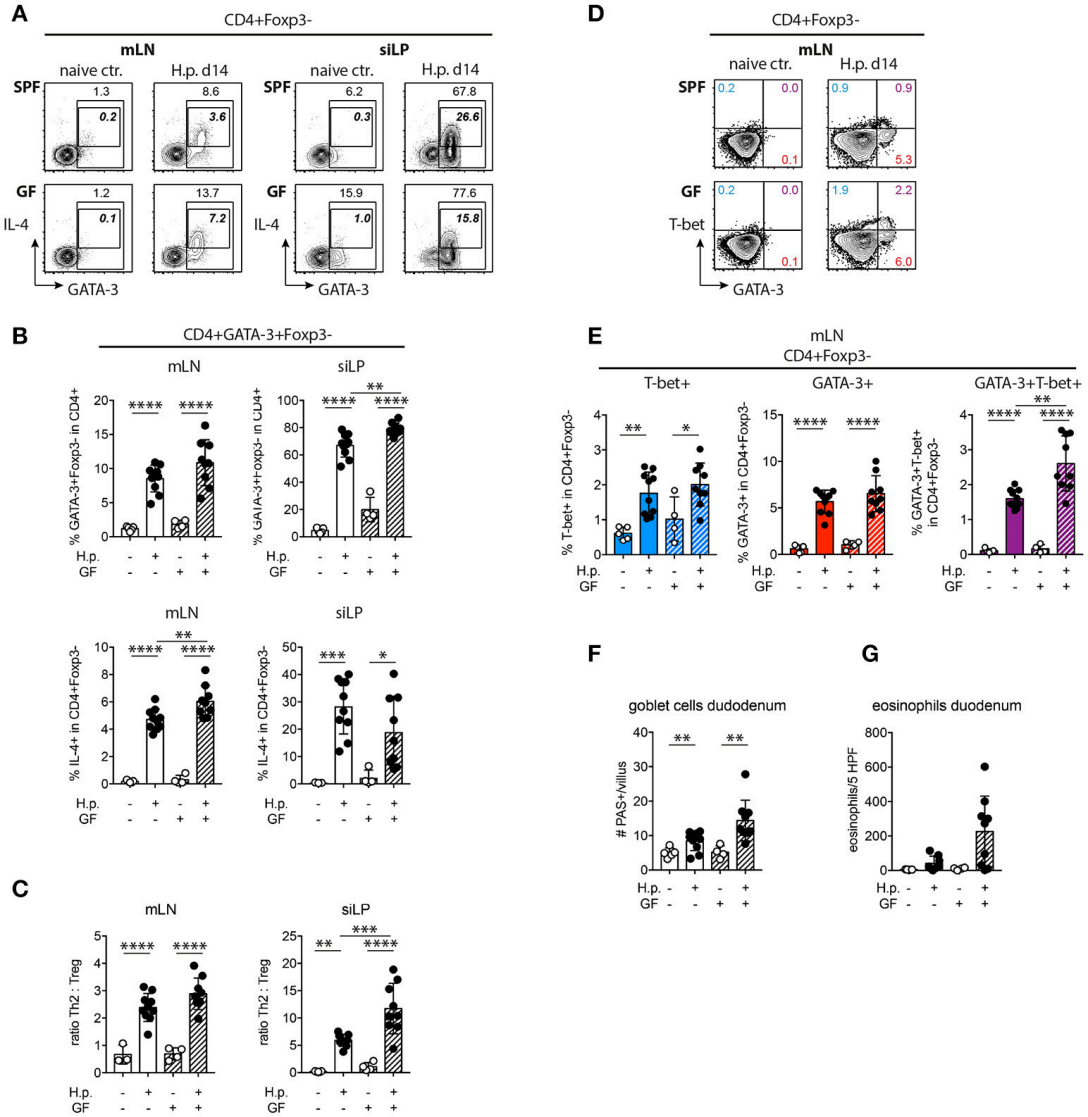
Th2 response and Th2/Treg ratios in SPF and GF mice. **(A,B)** Representative plots of GATA-3 and IL-4 expression by CD4^+^Foxp3^−^ T cells **(A)** and frequencies **(B)** of GATA-3^+^ and IL-4^+^ Th2 cells in mLN and siLP. Bold italic numbers in FACS plots refer to IL-4^+^ cells. **(C)** Ratios of GATA-3^+^ Th2 cells to Foxp3^+^ Treg in mLN and siLP determined based on frequencies in CD4^+^ T cells. **(D,E**) Representative plots of GATA-3 and T-bet expression by CD4^+^Foxp3^−^ T cells **(D)** and frequencies of T-bet^+^ Th1, GATA-3^+^ Th2, and GATA-3^+^T-bet^+^ Th2/1 cells in mLN **(E)**. **(F,G)** Histological goblet cell **(F)** and eosinophil **(G)** quantification in the small intestine of naïve and infected mice. Data are pooled from two independent experiments each performed with two to three uninfected and four to five infected mice per group. Mean, SD, and individual data points are shown. ^*^p < 0.05; ^**^p < 0.01, ^***^p < 0.001, ^****^p < 0.0001.

To see if the reduction in RORγt^+^ Treg and the lower IL-10 expression by small intestinal Treg in GF mice coincided with deregulated Th2 responses we quantified Th2 cells based on GATA-3 expression and IL-4 expression. Significantly more GATA-3^+^ Th2 cells were present in the small intestines of infected GF mice compared to SPF mice, while IL-4 production was significantly increased in mLN (Figures 5A,B). Calculating the ratios of Th2 cells to Treg based on their frequencies in CD4^+^ T cells, we found significantly elevated Th2 effector to Treg ratios in the small intestine of infected GF compared to SPF mice (Figure 5C). We have previously shown that intestinal nematode infections lead to the differentiation of GATA-3^+^ Th2 and GATA-3^+^T-bet^+^ Th2/1 hybrid cells (51, 52). Th2/1 cells developed in infected SPF as well as GF mic, hence microbial signals were dispensable for their induction (Figures 5D,E). The increase in intestinal GATA-3^+^Th2 cells coincided with trends of increased goblet cell and eosinophil counts in the duodenum (Figures 5F,G). As the microbiota supports Th17 differentiation (53, 54) we assessed RORγt and IL-17A expression by Foxp3^−^CD4^+^ T effector cells. Expectedly, GF mice harbored very few Th17 cells in mLN and small intestine (Figure S1). In conclusion, nematode-induced local Th2 responses were significantly increased in the absence of gut microbes and decreased parasite fitness in GF mice was associated with elevated Th2 to Treg ratios at the site of infection.

## Discussion

Over the last decade, several studies have shown that intestinal parasite infections lead to changes in the gut microbiota of the host [reviewed in (55, 56)]. Enteric nematodes such as *H. polygyrus, Nippostrongylus brasiliensis*, and *Trichuris* species alter the abundance of numerous bacterial genera in the host gut (2–4, 36, 57). Changes in the microbiota composition also result from infections with the protozoan parasites *Toxoplasma gondii* and *Giardia lamblia* (58, 59). Though the mechanistic basis for the microbiome changes provoked by the infections is not well understood, it is speculated that parasites may directly influence the composition of the microbiota. Parasite-driven immune responses resulting from tissue damage (3, 59) and leading to changes in gut physiology and epithelial barrier function (60–63) are likely to be involved. Nutrient competition and changes in host antimicrobial peptide production upon parasite infection may also contribute to structural changes in gut microbial communities (3, 64).

Here, we show that the excretory/secretory (E/S) products of the small intestinal nematode *H. polygyrus* exert antimicrobial activities seen as inhibited growth of several bacterial species including commensal intestinal species such as *E. faecium*, and agglutination of *E. coli*. Previous studies have reported on antimicrobial activity of nematode products, such as *Ascaris suum* antibacterial factors (ASABF) and cecropins (65, 66). We have recently shown that E/S products of the porcine roundworm *A. suum* possess antibacterial and agglutinating activity and impair biofilm formation (8). *Ascaris* E/S products comprise proteins and peptides with known and predicted antimicrobial activity, such as cecropins, ASABF, lysozymes, and C-type lectins (8). As our previous studies showed that changes in the gut microbiota of *H. polygyrus*-infected mice occurred independently of the parasite-driven Th2 response and subsequent changes in gut physiology (2), the detection of antimicrobial activities of nematode E/S products offers an attractive explanation of how these parasites may directly shape their microbial environment. On the other hand, strong Th2 responses, and the subsequent changes in host antimicrobial peptide and mucin production have been shown to be related to the decrease of segmented filamentous bacteria during infections with *N. brasiliensis* (3).

Of note, our previous studies showed an increase in Enterobacteriaceae along the small and large intestine upon infection with *H. polygyrus* (2). Whether the antimicrobial activity of *H. polygyrus* E/S products against Enterobacteriaceae family members such as *E. coli* and *S. enterica* prevents a more vigorous increase of such potentially pathogenic bacteria benefitting from intestinal inflammation can only be speculated on. It is conceivable that during coevolution, parasitic worms have not only developed intricate mechanisms interfering with host immunity, but also adapted to directly support or restrict the growth of commensal families which might be beneficial or detrimental to parasite survival and host health via the release of antimicrobial factors. Furthermore, the parasites may benefit from the support of immune regulatory circuits fostered by microbiome changes upon infection. Indeed, others have shown that *H. polygyrus* infection leads to the outgrowth of *Lactobacillus* species and members of the Clostridiales family, which in turn support the expansion and activation of regulatory T cells (1, 17). Our unpublished data show strong and selective antimicrobial activity of E/S products of *A. suum* on several members of the porcine microbiota, whereas *Clostridia* species displayed a growth advantage in presence of *A. suum* E/S. It hence seems that nematode infections provoke fine-tuned changes in the structure of the gut microbiome in favor of commensals supporting anti-inflammatory circuits, assisting host health and facilitating parasite survival. Future work will address if nematode antimicrobial factors such as cecropins, ASABF, lysozymes, and c-type lectins present in nematode E/S products differentially affect the growth of commensal and potentially pathogenic gut bacteria.

The release of antimicrobial factors by enteric nematodes and potential interference with the growth of certain bacterial species suggest that the parasites sense their microbial environment similar to free living worms such as *C. elegans* or *Pristionchus pacificus* (67, 68). However, whether intestinal parasites react by the differential expression of antimicrobial factors to environmental changes has not been assessed before. Here, we show that nematode gene expression is altered in the absence of host microbes. Our data provide evidence for microbial sensing by *H. polygyrus*, as factors with putative antimicrobial defense functions, such as chitinase (69) and lysozyme (70), were differentially expressed in nematodes isolated from germ-free in comparison to conventional mice, in addition to xenobiotic detoxification genes which are upregulated during bacterial infection of *C. elegans* (71). Interestingly, while lysozymes are thought to play an important role in nematode antimicrobial defenses (72), lysozyme-3 was upregulated in nematodes isolated from germ-free mice. Compared to worms reared in conventional mice, nematodes from germ-free mice develop in the face of a stronger Th2 response and are likely negatively impacted by the lack of a host microbiota, as evidenced by their reduced size and fecundity. Hence, upregulation of defense factors such as lysozyme-3 may be due to a stress response rather than a lack of microbial stimulation. This view is supported by the fact that also putative detoxification genes were upregulated in parasites isolated from germ-free mice. The altered gene expression of nematodes from germ-free mice might further result from the lack of microbial metabolic factors in the germ-free host gut. A direct dependence of *H. polygyrus* on small intestinal microbes as food source appears, however, unlikely, as host tissue, but not ingesta provide the main food source of the adult worms (42).

Several reports linked the host microbial status to differences in susceptibility for infections with intestinal helminths [reviewed in (56)]. We show here that *H. polygrus* adult worms display signs of reduced fitness when developing in GF mice, confirming early studies reporting impeded nematode infectivity and fitness in the absence of gut microbes (39–41). *H. polygyrus* fitness is determined by the magnitude of the anti-parasite Th2 response, evident as disparate worm fecundity and duration of infection in inbred mouse lines differing in Th2 reactivity (73). Anti-nematode immune responses are regulated by Treg, seen as increased Th2 and associated innate responses after Treg depletion, leading to lower worm burdens or shortened retention of adult worms in some experimental systems (12, 15, 74). Microbial signals are important for the activation and instruction of thymus-derived and peripherally induced Foxp3^+^ Treg in the gut (75). Here we show that the frequencies of Foxp3^+^ Treg were similar in conventional and GF mice infected with *H. polygyrus*, but the phenotypic composition of Foxp3^+^ Treg was altered in the small intestine and gut-associated lymphoid tissue of germ-free mice. Confirming a previous report (43), RORγt^+^Foxp3^+^ Treg were reduced in GF mice at steady state and after *H. polygyrus* infection, while the expansion of GATA-3^+^Foxp3^+^ Treg did not differ between infected SPF and GF mice. Whereas the complete absence of microbiota-induced RORgt^+^Foxp3^+^ Treg during *H. polygyrus* infection has been shown to result in the overt production of Th2 cytokines and reduced parasite fitness (43), our study provides evidence that more subtle changes in the intestinal Th2/Treg ratio are resulting from the germ-free status and, presumably, a reduction of microbiota-induced RORγt^+^Treg is sufficient to significantly stunt parasite fitness.

The production of IL-10 by regulatory T cells has been shown to be of central importance for the prevention of gut inflammation at steady state and in experimental settings of lung and skin inflammation (76). We show here that while IL-10 production by mLN-derived Treg increased significantly upon nematode infection irrespective of the host microbial status, IL-10 production by small intestinal Treg was not altered in response to infection. Furthermore, small intestinal Treg of GF mice displayed reduced IL-10 production at steady state and after nematode infection. The reduced IL-10 expression by small intestinal Treg of GF mice may in part be explained by the reduction in RORγt^+^ Treg, which have been previously reported as superior in IL-10 production compared to other intestinal Treg (43). Upon infection, however, we detected GATA-3^+^Treg as the dominant IL-10^+^ Treg source in the small intestine and mLN of SPF as well as GF mice. While our earlier studies have shown that Treg depletion during *H. polygyrus* infection results in increased small intestinal immunopathology (16), neither the decreased IL-10 production nor the reduction in RORγt^+^ Treg detected in nematode-infected GF mice reported here were associated with signs of increased gut inflammation.

In conclusion, the antimicrobial activity of nematode products reported here suggests that enteric helminths actively shape their microbial environment, possibly facilitating the outgrowth of microbes supporting immune regulatory circuits, and restricting the expansion of potentially harmful species. Our finding of stunted parasite fitness in germ-free mice associated with locally increased Th2 and blunted Treg responses is in line with previous reports on gut microbes affecting host susceptibility and Th2 reactivity during nematode infection. Future studies should assess if altering the gut microbiota could be used to shift the Th2/Treg balance in favor of parasite-specific effector cells and if parasite products may be employed to counteract states of pathological dysbiosis resulting from and perpetuating inflammation in intestinal inflammatory disorders.

## Data deposition

All sequencing data generated in this project are available from the NCBI Sequence Read Archive (SRA) and collectively available via the BioProject: PRJNA486010 and the SRA accession SRP157940, available at https://www.ncbi.nlm.nih.gov/bioproject/486010 and https://www.ncbi.nlm.nih.gov/sra/SRP157940.

## Author contributions

SH and SR conceptualized and designed the research. SR, AM, NA, and AR performed all the experiments. SR, AM, MK, NA, AR, AK and BR analyzed the data. SR, AM, MK, and SH wrote the manuscript. AR, AK, AB, and BR provided additional resources and edited the manuscript. All authors approved the final manuscript version.

### Conflict of interest statement

The authors declare that the research was conducted in the absence of any commercial or financial relationships that could be construed as a potential conflict of interest.

## References

[1] Zaiss MM Rapin A Lebon L Dubey LK Mosconi I Sarter K. The intestinal microbiota contributes to the ability of helminths to modulate allergic inflammation. Immunity (2015) 43:998–1010. 10.1016/j.immuni.2015.09.012 26522986 PMC4658337

[2] Rausch S Held J Fischer A Heimesaat MM Kühl AA Bereswill S. Small intestinal nematode infection of mice is associated with increased enterobacterial loads alongside the intestinal tract. PLoS ONE (2013) 8:e74026. 10.1371/journal.pone.0074026 24040152 PMC3769368

[3] Fricke WF Song Y Wang A-J Smith A Grinchuk V Pei C Type 2 immunity-dependent reduction of segmented filamentous bacteria in mice infected with the helminthic parasite *Nippostrongylus brasiliensis*. Microbiome (2015) 3:40 10.1186/s40168-015-0103-8 26377648 PMC4574229

[4] Li RW Wu S Li W Navarro K Couch RD Hill D. Alterations in the porcine colon microbiota induced by the gastrointestinal nematode *Trichuris suis*. Infect Immun. (2012) 80:2150–7. 10.1128/IAI.00141-12 22493085 PMC3370577

[5] Broadhurst MJ Ardeshir A Kanwar B Mirpuri J Gundra UM Leung JM. Therapeutic helminth infection of macaques with idiopathic chronic diarrhea alters the inflammatory signature and mucosal microbiota of the colon. PLoS Pathog. (2012) 8:e1003000. 10.1371/journal.ppat.1003000 23166490 PMC3499566

[6] Abner SR Parthasarathy G Hill DE Mansfield LS. *Trichuris suis*: detection of antibacterial activity in excretory-secretory products from adults. Exp Parasitol. (2001) 99:26–36. 10.1006/expr.2001.4643 11708831

[7] Midha A Schlosser J Hartmann S. Reciprocal interactions between nematodes and their microbial environments. Front. Cell. Infect. Microbiol. (2017) 7:144. 10.3389/fcimb.2017.00144 28497029 PMC5406411

[8] Midha A Janek K Niewienda A Henklein P Guenther S Serra DO. The intestinal roundworm ascaris suum releases antimicrobial factors which interfere with bacterial growth and biofilm formation. Front. Cell. Infect. Microbiol. (2018) 8:271. 10.3389/fcimb.2018.00271. 30131945 PMC6090379

[9] Maizels RM Hewitson JP Murray J Harcus YM Dayer B Filbey KJ. Immune modulation and modulators in *Heligmosomoides polygyrus* infection. Exp Parasitol. (2012) 132:76–89. 10.1016/j.exppara.2011.08.011 21875581 PMC6485391

[10] Maizels RM McSorley HJ. Regulation of the host immune system by helminth parasites. J Allergy Clin Immunol. (2016) 138:666–75. 10.1016/j.jaci.2016.07.007 27476889 PMC5010150

[11] Ziegler T Rausch S Steinfelder S Klotz C Hepworth MR Kühl AA. A novel regulatory macrophage induced by a helminth molecule instructs IL-10 in CD4+ T cells and protects against mucosal inflammation. J Immunol. (2015) 194:1555–64. 10.4049/jimmunol.1401217 25589067

[12] Blankenhaus B Reitz M Brenz Y Eschbach M-L Hartmann W Haben I Foxp3+ regulatory T cells delay expulsion of intestinal nematodes by suppression of IL-9-driven mast cell activation in BALB/c but not in C57BL/6 mice. PLoS Pathog. (2014) 10:e1003913 10.1371/journal.ppat.1003913 24516385 PMC3916398

[13] Finney CAM Taylor MD Wilson MS Maizels RM. Expansion and activation of CD4+CD25+ regulatory T cells in *Heligmosomoides polygyrus* infection. Eur J Immunol. (2007) 37:1874–86. 10.1002/eji.200636751 17563918 PMC2699425

[14] Grainger JR Smith KA Hewitson JP McSorley HJ Harcus Y Filbey KJ. Helminth secretions induce de novo T cell Foxp3 expression and regulatory function through the TGF-β pathway. J Exp Med. (2010) 207:2331–41. 10.1084/jem.20101074 20876311 PMC2964568

[15] Rausch S Huehn J Kirchhoff D Rzepecka J Schnoeller C Pillai S. Functional analysis of effector and regulatory T cells in a parasitic nematode infection. Infect Immun. (2008) 76:1908–19. 10.1128/IAI.01233-07 18316386 PMC2346705

[16] Rausch S Huehn J Loddenkemper C Hepworth MR Klotz C Sparwasser T. Establishment of nematode infection despite increased Th2 responses and immunopathology after selective depletion of Foxp3+ cells. Eur. J. Immunol. (2009) 39:3066–77. 10.1002/eji.200939644 19750483

[17] Reynolds LA Smith KA Filbey KJ Harcus Y Hewitson JP Redpath SA. Commensal-pathogen interactions in the intestinal tract. Gut Microb. (2014) 5:522–32. 10.4161/gmic.32155 25144609 PMC4822684

[18] Wilson MS Taylor MD Balic A Finney CAM Lamb JR Maizels RM. Suppression of allergic airway inflammation by helminth-induced regulatory T cells. J Exp Med. (2005) 202:1199–212. 10.1084/jem.20042572 16275759 PMC2213237

[19] Takemura H Kaku M Kohno S Hirakata Y Tanaka H Yoshida R. Evaluation of susceptibility of gram-positive and -negative bacteria to human defensins by using radial diffusion assay. Antimicrob Agents Chemother. (1996) 40:2280–4. 8891130 10.1128/aac.40.10.2280 PMC163519

[20] Gasmi L Ferré J Herrero S. High bacterial agglutination activity in a single-CRD C-type lectin from *Spodoptera exigua* (Lepidoptera: Noctuidae). Biosensors (2017) 7:12. 10.3390/bios7010012 28257054 PMC5371785

[21] Bolger AM Lohse M Usadel B. Trimmomatic: a flexible trimmer for Illumina sequence data. Bioinformatics (2014) 30:2114–20. 10.1093/bioinformatics/btu170 24695404 PMC4103590

[22] Wood DE Salzberg SL. Kraken: ultrafast metagenomic sequence classification using exact alignments. Genome Biol. (2014) 15:R46. 10.1186/gb-2014-15-3-r46 24580807 PMC4053813

[23] Kim D Pertea G Trapnell C Pimentel H Kelley R Salzberg SL. TopHat2: accurate alignment of transcriptomes in the presence of insertions, deletions and gene fusions. Genome Biol. (2013) 14:R36. 10.1186/gb-2013-14-4-r36 23618408 PMC4053844

[24] Trapnell C Hendrickson DG Sauvageau M Goff L Rinn JL Pachter L. Differential analysis of gene regulation at transcript resolution with RNA-seq. Nat Biotechnol. (2013) 31:46–53. 10.1038/nbt.2450 23222703 PMC3869392

[25] Howe KL Bolt BJ Shafie M Kersey P Berriman M. WormBase ParaSite – a comprehensive resource for helminth genomics. Mol Biochem Parasitol. (2017) 215:2–10. 10.1016/j.molbiopara.2016.11.005 27899279 PMC5486357

[26] Liao Y Smyth GK Shi W. featureCounts: an efficient general purpose program for assigning sequence reads to genomic features. Bioinformatics (2014) 30:923–30. 10.1093/bioinformatics/btt656 24227677

[27] Love MI Huber W Anders S. Moderated estimation of fold change and dispersion for RNA-seq data with DESeq2. Genome Biol. (2014) 15:550. 10.1186/s13059-014-0550-8 25516281 PMC4302049

[28] Ritchie ME Phipson B Wu D Hu Y Law CW Shi W. limma powers differential expression analyses for RNA-sequencing and microarray studies. Nucleic Acids Res. (2015) 43:e47. 10.1093/nar/gkv007 25605792 PMC4402510

[29] Rice P Longden I Bleasby A. EMBOSS: the European molecular biology open software suite. Trends Genet. (2000) 16:276–7. 10.1016/S0168-9525(00)02024-2 10827456

[30] Ashburner M Ball CA Blake JA Botstein D Butler H Cherry JM. Gene ontology: tool for the unification of biology. Nat. Genet. (2000) 25:25–9. 10.1038/75556 10802651 PMC3037419

[31] Camacho C Coulouris G Avagyan V Ma N Papadopoulos J Bealer K. BLAST+: architecture and applications. BMC Bioinformatics (2009) 10:421. 10.1186/1471-2105-10-421 20003500 PMC2803857

[32] Conesa A Götz S García-Gómez JM Terol J Talón M Robles M. Blast2GO: a universal tool for annotation, visualization and analysis in functional genomics research. Bioinformatics (2005) 21:3674–6. 10.1093/bioinformatics/bti610 16081474

[33] Jones P Binns D Chang H-Y Fraser M Li W McAnulla C. InterProScan 5: genome-scale protein function classification. Bioinformatics (2014) 30:1236–40. 10.1093/bioinformatics/btu031 24451626 PMC3998142

[34] The UniProt Consortium (2017). UniProt: the universal protein knowledgebase. Nucleic Acids Res. 45:D158–69. 27899622 10.1093/nar/gkw1099 PMC5210571

[35] Strandmark J Steinfelder S Berek C Kühl AA Rausch S Hartmann S. Eosinophils are required to suppress Th2 responses in Peyer's patches during intestinal infection by nematodes. Mucosal Immunol. (2017) 10:661–72. 10.1093/nar/gkh131 27805618

[36] Walk ST Blum AM Ewing SA-S Weinstock JV Young VB. Alteration of the murine gut microbiota during infection with the parasitic helminth *Heligmosomoides polygyrus*. Inflamm Bowel Dis. (2010) 16:1841–9. 10.1002/ibd.21299 20848461 PMC2959136

[37] Miltsch SM Seeberger PH Lepenies B. The C-type lectin-like domain containing proteins Clec-39 and Clec-49 are crucial for *Caenorhabditis elegans* immunity against *Serratia marcescens* infection. Dev Comp Immunol. (2014) 45:67–73. 10.1016/j.dci.2014.02.002 24534554

[38] Harcus Y Nicoll G Murray J Filbey K Gomez-Escobar N Maizels RM. C-type lectins from the nematode parasites *Heligmosomoides polygyrus* and *Nippostrongylus brasiliensis*. Parasitol Int. (2009) 58:461–70. 10.1016/j.parint.2009.08.011 19751847 PMC2792708

[39] Chang J Wescott RB. Infectivity, fecundity, and survival of Nematospiroides dubius in gnotobiotic mice. Exp Parasitol. (1972) 32:327–34. 10.1016/0014-4894(72)90060-4 4675136

[40] Wescott RB. Experimental Nematospiroides dubius infection in germfree and conventional mice. Exp Parasitol. (1968) 22:245–9. 10.1016/0014-4894(68)90099-4 5652501

[41] Wescott RB Todd AC. A Comparison of the development of *Nippostrongylus brasiliensis* in germ-free and conventional mice. J Parasitol. (1964) 50:138–43. 10.2307/3276048 14125156

[42] Bansemir AD Sukhdeo MV. The food resource of adult *Heligmosomoides polygyrus* in the small intestine. J Parasitol. (1994) 80:24–8. 10.2307/3283340 8308654

[43] Ohnmacht C Park J-H Cording S Wing JB Atarashi K Obata Y. The microbiota regulates type 2 immunity through RORγt^+^ T cells. Science (2015) 349:989–93. 10.1126/science.aac4263 26160380

[44] Smith PM Howitt MR Panikov N Michaud M Gallini CA Bohlooly YM. The microbial metabolites, short-chain fatty acids, regulate colonic treg cell homeostasis. Science (2013) 341:569–73. 10.1126/science.1241165 23828891 PMC3807819

[45] Round JL Mazmanian SK. Inducible Foxp3+ regulatory T-cell development by a commensal bacterium of the intestinal microbiota. Proc Natl Acad Sci USA. (2010) 107:12204–9. 10.1073/pnas.0909122107 20566854 PMC2901479

[46] Korn LL Hubbeling HG Porrett PM Yang Q Barnett LG Laufer TM. Regulatory T cells occupy an isolated niche in the intestine that is antigen independent. Cell Rep. (2014) 9:1567–73. 10.1016/j.celrep.2014.11.006 25482559 PMC12079782

[47] Luu M Steinhoff U Visekruna A. Functional heterogeneity of gut-resident regulatory T cells. Clin Transl Immunol. (2017) 6:e156. 10.1038/cti.2017.39 28983404 PMC5628268

[48] Wang Y Su MA Wan YY. An essential role of the transcription factor GATA-3 for the function of regulatory t cells. Immunity (2011) 35:337–48. 10.1016/j.immuni.2011.08.012 21924928 PMC3182399

[49] Wohlfert EA Grainger JR Bouladoux N Konkel JE Oldenhove G Ribeiro CH. GATA3 controls Foxp3^+^ regulatory T cell fate during inflammation in mice. J Clin Invest. (2011) 121:4503–15. 10.1172/JCI57456 21965331 PMC3204837

[50] Yang B-H Hagemann S Mamareli P Lauer U Hoffmann U Beckstette M. Foxp3^+^ T cells expressing RORγt represent a stable regulatory T-cell effector lineage with enhanced suppressive capacity during intestinal inflammation. Mucosal Immunol. (2016) 9:444–57. 10.1038/mi.2015.74 26307665

[51] Bock CN Babu S Breloer M Rajamanickam A Boothra Y Brunn M-L. Th2/1 hybrid cells occurring in murine and human strongyloidiasis share effector functions of Th1 cells. Front. Cell. Infect. Microbiol. (2017) 7:261. 10.3389/fcimb.2017.00261 28676845 PMC5476698

[52] Peine M Rausch S Helmstetter C Fröhlich A Hegazy AN Kühl AA. Stable T-bet(+)GATA-3(+) Th1/Th2 hybrid cells arise *in vivo*, can develop directly from naive precursors, and limit immunopathologic inflammation. PLoS Biol. (2013) 11:e1001633. 10.1371/journal.pbio.1001633 23976880 PMC3747991

[53] Atarashi K Tanoue T Ando M Kamada N Nagano Y Narushima S. Th17 cell induction by adhesion of microbes to intestinal epithelial cells. Cell (2015) 163:367–80. 10.1016/j.cell.2015.08.058 26411289 PMC4765954

[54] Ivanov II Atarashi K Manel N Brodie EL Shima T Karaoz U. Induction of intestinal Th17 cells by segmented filamentous bacteria. Cell (2009) 139:485–98. 10.1016/j.cell.2009.09.033 19836068 PMC2796826

[55] Burgess SL Gilchrist CA Lynn TC Petri WA. Parasitic protozoa and interactions with the host intestinal microbiota. Infect Immun. (2017) 85:e00101–17. 10.1128/IAI.00101-17 28584161 PMC5520446

[56] Zaiss MM Harris NL. Interactions between the intestinal microbiome and helminth parasites. Parasite Immunol. (2016) 38:5–11. 10.1111/pim.12274 26345715 PMC5019230

[57] Holm JB Sorobetea D Kiilerich P Ramayo-Caldas Y Estellé J Ma T. Chronic trichuris muris infection decreases diversity of the intestinal microbiota and concomitantly increases the abundance of Lactobacilli. PLOS ONE (2015) 10:e0125495. 10.1371/journal.pone.0125495 25942314 PMC4420551

[58] Barash NR Maloney JG Singer SM Dawson SC. Giardia alters commensal microbial diversity throughout the murine gut. Infect Immun. (2017) 85:e00948–16. 10.1128/IAI.00948-16 28396324 PMC5442636

[59] Heimesaat MM Bereswill S Fischer A Fuchs D Struck D Niebergall J. Gram-negative bacteria aggravate murine small intestinal Th1-type immunopathology following oral infection with *Toxoplasma gondii*. J Immunol. (2006) 177:8785–95. 10.4049/jimmunol.177.12.8785 17142781

[60] Zhao A McDermott J Urban JF Gause W Madden KB Yeung KA. Dependence of IL-4, IL-13, and nematode-induced alterations in murine small intestinal smooth muscle contractility on Stat6 and enteric nerves. J Immunol. (2003) 171:948–54. 10.4049/jimmunol.171.2.948 12847266

[61] Marillier RG Michels C Smith EM Fick LC Leeto M Dewals B. IL-4/IL-13 independent goblet cell hyperplasia in experimental helminth infections. BMC Immunol. (2008) 9:11. 10.1186/1471-2172-9-11 18373844 PMC2329604

[62] McKay DM Shute A Lopes F. Helminths and intestinal barrier function. Tissue Barr. (2017) 5:e1283385. 10.1080/21688370.2017.1283385 28452686 PMC5362995

[63] Finkelman FD Shea-Donohue T Morris SC Gildea L Strait R Madden KB. Interleukin-4- and interleukin-13-mediated host protection against intestinal nematode parasites. Immunol. Rev. (2004) 201:139–55. 10.1111/j.0105-2896.2004.00192.x 15361238

[64] Manko A Motta J-P Cotton JA Feener T Oyeyemi A Vallance BA. Giardia co-infection promotes the secretion of antimicrobial peptides beta-defensin 2 and trefoil factor 3 and attenuates attaching and effacing bacteria-induced intestinal disease. PLOS ONE (2017) 12:e0178647. 10.1371/journal.pone.0178647 28622393 PMC5473565

[65] Pillai A Ueno S Zhang H Kato Y. Induction of ASABF (*Ascaris suum* antibacterial factor)-type antimicrobial peptides by bacterial injection: novel members of ASABF in the nematode *Ascaris suum*. Biochem J. (2003) 371:663–8. 10.1042/bj20021948 12617723 PMC1223358

[66] Pillai A Ueno S Zhang H Lee JM Kato Y. Cecropin P1 and novel nematode cecropins: a bacteria-inducible antimicrobial peptide family in the nematode *Ascaris suum*. Biochem J. (2005) 390:207–14. 10.1042/BJ20050218 15850460 PMC1184576

[67] Samuel BS Rowedder H Braendle C Félix M-A Ruvkun G. *Caenorhabditis elegans* responses to bacteria from its natural habitats. Proc Natl Acad Sci USA. (2016) 113:E3941–9. 10.1073/pnas.1607183113 27317746 PMC4941482

[68] Sinha A Rae R Iatsenko I Sommer RJ. System wide analysis of the evolution of innate immunity in the nematode model species *Caenorhabditis elegans* and *Pristionchus pacificus*. PLoS ONE (2012) 7:e44255. 10.1371/journal.pone.0044255 23028509 PMC3461006

[69] Chung MC Dean S Marakasova ES Nwabueze AO van Hoek ML. Chitinases are negative regulators of *Francisella novicida* biofilms. PLoS ONE (2014) 9:e93119. 10.1371/journal.pone.0093119 24664176 PMC3963990

[70] Dierking K Yang W Schulenburg H. Antimicrobial effectors in the nematode *Caenorhabditis elegans*: an outgroup to the Arthropoda. Philos Trans R Soc B Biol Sci. (2016) 371:20150299. 10.1098/rstb.2015.0299 27160601 PMC4874396

[71] Pukkila-Worley R. Surveillance immunity: an emerging paradigm of innate defense activation in *Caenorhabditis elegans*. PLOS Pathog. (2016) 12:e1005795. 10.1371/journal.ppat.1005795 27631629 PMC5025020

[72] Schulenburg H Boehnisch C. Diversification and adaptive sequence evolution of Caenorhabditislysozymes (Nematoda: Rhabditidae). BMC Evol Biol. (2008) 8:114. 10.1186/1471-2148-8-114 18423043 PMC2383907

[73] Filbey KJ Grainger JR Smith KA Boon L van Rooijen N Harcus Y. Innate and adaptive type 2 immune cell responses in genetically controlled resistance to intestinal helminth infection. Immunol Cell Biol. (2014) 92:436–48. 10.1038/icb.2013.109 24492801 PMC4038150

[74] Smith KA Filbey KJ Reynolds LA Hewitson JP Harcus Y Boon L. Low-level regulatory T-cell activity is essential for functional type-2 effector immunity to expel gastrointestinal helminths. Mucosal Immunol. (2016) 9:428–43. 10.1038/mi.2015.73 26286232 PMC4677460

[75] Geuking MB Cahenzli J Lawson MAE Ng DCK Slack E Hapfelmeier S. Intestinal bacterial colonization induces mutualistic regulatory T cell responses. Immunity (2011) 34:794–806. 10.1016/j.immuni.2011.03.021 21596591

[76] Rubtsov YP Rasmussen JP Chi EY Fontenot J Castelli L Ye X. Regulatory T cell-derived interleukin-10 limits inflammation at environmental interfaces. Immunity (2008) 28:546–58. 10.1016/j.immuni.2008.02.017 18387831

