# Supplementary material for: Parasitic Nematodes Exert Antimicrobial Activity and Benefit From Microbiota-Driven Support for Host Immune Regulation
Source: Front Immunol. 2018 Oct 8;9:2282. doi: 10.3389/fimmu.2018.02282 (PMC6186814; doi:10.3389/fimmu.2018.02282)
Supplement: Supplementary file 1 [file Table_1.DOCX]

Rausch et al.

**Supplementary Table 1. Significantly* differentially expressed genes (DEGs) of *H. polygyrus* isolated from germ-free mice compared to SPF isolates.**

| **Gene ID** | **Sequence description** | **padj** | **Fold change** |
| --- | --- | --- | --- |
| gene_HPOL_0000075301 | Uncharacterized protein OS=Heligmosomoides polygyrus bakeri PE=4 SV=1 | 6,28E-18 | 3,844818179 |
| gene_HPOL_0002275401 | Uncharacterized protein OS=Heligmosomoides polygyrus bakeri PE=4 SV=1 | 3,14E-12 | 2,969487596 |
| gene_HPOL_0000075201 | Venom allergen/ancylostoma secreted protein-like 1 isoform 3 OS=Heligmosomoides bakeri PE=4 SV=1 | 7,72E-08 | 2,697561657 |
| gene_HPOL_0002228701 | Uncharacterized protein OS=Heligmosomoides polygyrus bakeri PE=4 SV=1 | 4,95E-05 | 2,262499456 |
| gene_HPOL_0001072601 | Complement factor H OS=Bos taurus GN=CFH PE=1 SV=3 | 6,99E-06 | 2,181984829 |
| gene_HPOL_0002090901 | NA | 0,000319199 | 2,117700656 |
| gene_HPOL_0000515201 | Uncharacterized protein OS=Heligmosomoides polygyrus bakeri PE=4 SV=1 | 1,91E-05 | 2,092571673 |
| gene_HPOL_0001104101 | Uncharacterized protein OS=Heligmosomoides polygyrus bakeri PE=4 SV=1 | 0,000255441 | 2,019353057 |
| gene_HPOL_0001228401 | Uncharacterized protein OS=Heligmosomoides polygyrus bakeri PE=4 SV=1 | 0,00158136 | 2,000836347 |
| gene_HPOL_0000515801 | Uncharacterized protein OS=Heligmosomoides polygyrus bakeri PE=4 SV=1 | 0,002232693 | 1,977192422 |
| gene_HPOL_0001253401 | Chitinase-1 OS=Heligmosomoides bakeri GN=chi-1 PE=2 SV=1 | 8,67E-09 | 1,95111089 |
| gene_HPOL_0001276301 | CBN-GLB-9 protein OS=Haemonchus contortus GN=HCOI_00308200 PE=4 SV=1 | 0,00628251 | 1,932731756 |
| gene_HPOL_0000534501 | Uncharacterized protein OS=Heligmosomoides polygyrus bakeri PE=4 SV=1 | 6,97E-05 | 1,921169338 |
| gene_HPOL_0000224901 | Uncharacterized protein OS=Heligmosomoides polygyrus bakeri PE=4 SV=1 | 0,004696443 | 1,916382906 |
| gene_HPOL_0001274901 | Uncharacterized protein OS=Heligmosomoides polygyrus bakeri PE=4 SV=1 | 0,014792395 | 1,861349077 |
| gene_HPOL_0001491401 | Phospholipase A2 domain [InterPro - Gene3D,SUPERFAMILY] | 0,00113608 | 1,843006978 |
| XLOC_015764 | NA | 0,000307361 | 1,839373261 |
| gene_HPOL_0001712301 | Uncharacterized protein OS=Heligmosomoides polygyrus bakeri PE=4 SV=1 | 0,000143899 | 1,831882426 |
| gene_HPOL_0001805501 | Uncharacterized protein OS=Ancylostoma ceylanicum GN=ANCCEY_10701 PE=4 SV=1 | 0,015829617 | 1,814696853 |
| gene_HPOL_0001856901 | Uncharacterized protein OS=Heligmosomoides polygyrus bakeri PE=4 SV=1 | 0,021992714 | 1,812942965 |
| gene_HPOL_0002062301 | Uncharacterized protein OS=Heligmosomoides polygyrus bakeri PE=4 SV=1 | 0,000436217 | 1,81220383 |
| XLOC_010618 | NA | 0,021992714 | 1,812044524 |
| gene_HPOL_0000759301 | DB module OS=Ancylostoma ceylanicum GN=ANCCEY_06369 PE=4 SV=1 | 0,034989613 | 1,789867766 |
| gene_HPOL_0001350401 | Uncharacterized protein OS=Heligmosomoides polygyrus bakeri PE=4 SV=1 | 0,048233311 | 1,759308851 |
| gene_HPOL_0000843101 | Uncharacterized protein OS=Heligmosomoides polygyrus bakeri PE=4 SV=1 | 0,006573203 | 1,757225777 |
| gene_HPOL_0001875501 | 3-hydroxyacyl-CoA dehydrogenase domain containing protein OS=Haemonchus contortus GN=HCOI_02142700 PE=4 SV=1 | 0,027727141 | 1,754425341 |
| gene_HPOL_0000187401 | Ancylostoma secreted protein OS=Ancylostoma caninum GN=ASP PE=2 SV=1 | 0,010787242 | 1,749585723 |
| gene_HPOL_0002109201 | Uncharacterized protein OS=Heligmosomoides polygyrus bakeri PE=4 SV=1 | 0,04978098 | 1,744865558 |
| gene_HPOL_0001859101 | Probable glutathione S-transferase 8 OS=Caenorhabditis elegans GN=gst-8 PE=3 SV=1 | 0,006534013 | 1,732608938 |
| XLOC_001690 | NA | 0,002232693 | 1,724168184 |
| gene_HPOL_0001663001 | Coagulation factor XIII B chain OS=Homo sapiens GN=F13B PE=1 SV=3 | 0,003094029 | 1,723248042 |
| gene_HPOL_0000311101 | Putative uncharacterized protein OS=Haemonchus contortus PE=2 SV=1 | 0,00051066 | 1,721278816 |
| gene_HPOL_0001833801 | Uncharacterized protein OS=Heligmosomoides polygyrus bakeri PE=4 SV=1 | 0,016438884 | 1,70903542 |
| gene_HPOL_0000515001 | PHA domain OS=Heligmosomoides bakeri GN=php-1 PE=2 SV=1 | 0,027029698 | 1,704782436 |
| gene_HPOL_0001811801 | Lysozyme-3 OS=Heligmosomoides bakeri GN=lys-3 PE=2 SV=1 | 0,005070801 | 1,679111105 |
| gene_HPOL_0000747901 | Uncharacterized protein OS=Heligmosomoides polygyrus bakeri PE=4 SV=1 | 0,004261855 | 1,66974639 |
| gene_HPOL_0000467601 | K+ channel tetramerisation domain protein OS=Ancylostoma ceylanicum GN=Acey_s0060.g3129 PE=4 SV=1 | 0,006775472 | 1,660379443 |
| gene_HPOL_0001347701 | Uncharacterized protein OS=Heligmosomoides polygyrus bakeri PE=4 SV=1 | 0,017540267 | 1,651066821 |
| gene_HPOL_0001267301 | Delta l-pyrroline-5-carboxylate synthetase (Fragment) OS=Necator americanus GN=NECAME_01073 PE=3 SV=1 | 0,014792395 | 1,642245034 |
| gene_HPOL_0001166301 | NA | 0,041913733 | 1,586916529 |
| gene_HPOL_0001027001 | Putative UDP-glucuronosyltransferase ugt-47 OS=Caenorhabditis briggsae GN=ugt-47 PE=3 SV=3 | 0,014792395 | 1,543983397 |
| gene_HPOL_0000759601 | 4-hydroxyphenylpyruvate dioxygenase OS=Caenorhabditis elegans GN=hpd-1 PE=3 SV=1 | 0,04978098 | 1,533408979 |
| gene_HPOL_0001917901 | Channel protein, MIP family OS=Necator americanus GN=NECAME_00618 PE=3 SV=1 | 0,014792395 | 0,659284267 |
| gene_HPOL_0001711001 | Metalloendopeptidase OS=Heligmosomoides bakeri PE=3 SV=1 | 0,014792395 | 0,645189916 |
| gene_HPOL_0002173501 | Putative UDP-glucuronosyltransferase ugt-56 OS=Caenorhabditis elegans GN=ugt-56 PE=3 SV=2 | 0,036027788 | 0,643079489 |
| gene_HPOL_0002210401 | Uncharacterized protein OS=Heligmosomoides polygyrus bakeri PE=4 SV=1 | 0,04978098 | 0,594789839 |
| gene_HPOL_0002210201 | Uncharacterized protein OS=Heligmosomoides polygyrus bakeri PE=4 SV=1 | 0,013888521 | 0,576631292 |
| XLOC_009471 | NA | 0,00628251 | 0,553438681 |
| XLOC_019668 | NA | 0,010210278 | 0,547923657 |
| XLOC_025474 | Uncharacterized protein OS=Heligmosomoides polygyrus bakeri PE=4 SV=1 | 0,000195202 | 0,505709733 |
| XLOC_000542 | NA | 0,001573513 | 0,491777702 |
| gene_HPOL_0001560001 | Pyridoxal phosphate-dependent decarboxylase domain containing protein OS=Haemonchus contortus GN=HCOI_01605400 PE=3 SV=1 | 7,20E-19 | 0,235490448 |

_*_ padj: differential expression p-value according to DESeq2, including Benjamini-Hochberg adjustment for multiple testing.

Upregulated genes are marked in green, downregulated genes are marked in red.
