# Supplementary figures and images for: Parasitic Nematodes Exert Antimicrobial Activity and Benefit From Microbiota-Driven Support for Host Immune Regulation
Source: Front Immunol. 2018 Oct 8;9:2282. doi: 10.3389/fimmu.2018.02282 (PMC6186814; doi:10.3389/fimmu.2018.02282)

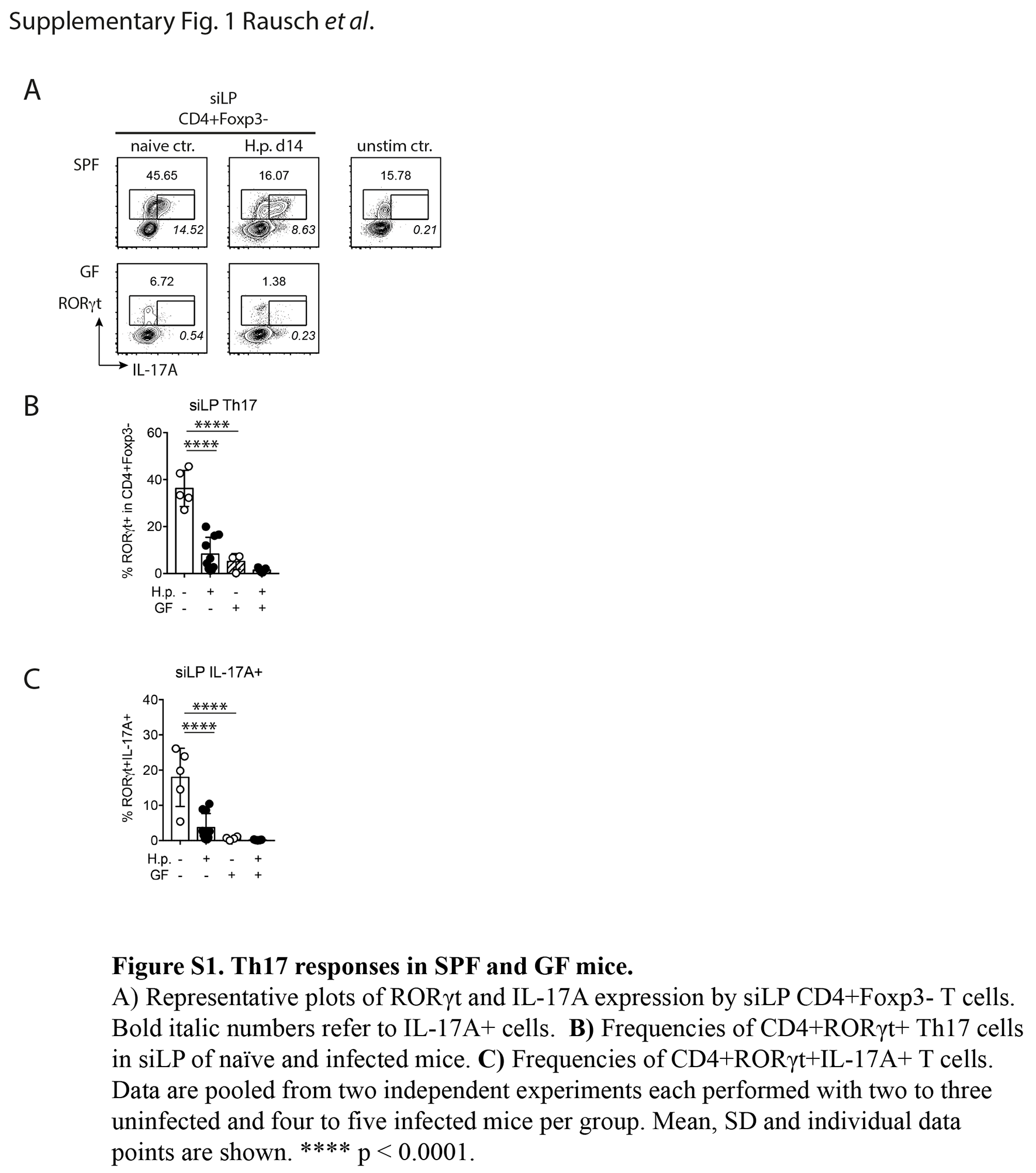

Supplement: Supplementary file 2 [file Image_1.TIF]
